# Characteristics of a Canine Distemper Virus Outbreak in Dichato, Chile Following the February 2010 Earthquake

**DOI:** 10.3390/ani3030843

**Published:** 2013-08-27

**Authors:** Elena Garde, Guillermo Pérez, Gerardo Acosta-Jamett, Barend Mark Bronsvoort

**Affiliations:** 1 Division of Pathway Medicine, School of Biomedical Sciences, College of Medicine and Veterinary Medicine, The University of Edinburgh, Edinburgh, EH16 4SB, UK; 2 Latin America Branch, Veterinarians Without Borders (Veterinarios Sin Fronteras) Canada, Pasaje Los Arrayanes 333, Valdivia, Chile; E-Mail: guillermo@vetswithoutborders.ca; 3 Instituto de Medicina Preventiva Veterinaria, Facultad de Ciencias Veterinarias, Universidad Austral de Chile, Casilla 567, Valdivia, Chile; E-Mail: gerardo.acosta@uach.cl; 4 The Roslin Institute and Royal (Dick) School of Veterinary Studies, University of Edinburgh, Midlothian, EH25 9RG, UK; E-Mail: mark.bronsvoort@roslin.ed.ac.uk

**Keywords:** free-roaming dogs, Chile, canine distemper virus, disaster preparedness, natural disaster, companion animals, disaster response

## Abstract

**Simple Summary:**

A disease outbreak in domestic dogs was reported by a coastal Chilean community following the February 2010 earthquake and tsunami. Using clinical exams and diagnostic testing, canine distemper virus was confirmed. Most dogs seen had never been vaccinated, and the majority of those with positive results were recorded in dogs less than two years of age. There were no facilities to contain or treat dogs locally, and no plan to shelter free-roaming dogs. This observational study demonstrates the great need for disaster preparedness planning in developing countries that includes companion animals.

**Abstract:**

Following the earthquake and tsunami disaster in Chile in February 2010, residents of Dichato reported high morbidity and mortality in dogs, descriptions of which resembled canine distemper virus (CDV). To assess the situation, free vaccine clinics were offered in April and May. Owner information, dog history and signalment were gathered; dogs received physical examinations and vaccines protecting against CDV, and other common canine pathogens. Blood was collected to screen for IgM antibodies to CDV. In total, 208 dogs received physical exams and vaccines were given to 177. IgM antibody titres to CDV were obtained for 104 dogs. Fifty-four dogs (51.9%) tested positive for CDV at the cut off titre of >1:50, but a total of 91.4% of dogs had a detectable titre >1:10. Most of the positive test results were in dogs less than 2 years of age; 33.5% had been previously vaccinated against CDV, and owners of 84 dogs (42.2%) reported clinical signs characteristic of CDV in their dogs following the disaster. The presence of endemic diseases in dog populations together with poor pre-disaster free-roaming dog management results in a potential for widespread negative effects following disasters. Creation of preparedness plans that include animal welfare, disease prevention and mitigation should be developed.

## 1. Introduction

Canine distemper virus (CDV) is one of the most important and devastating infectious diseases of canines, and is increasing in incidence around the world [1,2,3]. However, little is known about the potential for outbreaks following disasters. In an age where disasters are on the rise [4], and with over 500 million dogs [5] currently estimated worldwide, a disease as contagious and lethal as CDV warrants special attention, particularly in the free-roaming population that seldom receives veterinary attention [6].

In developing countries around the world, free-roaming dogs (FRDs) are a common problem [7], and CDV vaccination rates are generally too low to provide adequate herd immunity [6,8,9]. In urban areas where FRDs are present at high densities, they have the ability to maintain CDV within their population, presenting significant risks to themselves and other susceptible species [8]. Dramatic environmental alterations, such as those seen following natural disasters, are stressful events [10], and could be sufficient to precipitate outbreaks of endemic diseases such as CDV in FRDs [6,11,12]. 

CDV is a highly contagious, acute multi-systemic infection caused by a virus of the genus *Morbillivirus*. Signs include loss of appetite, biphasic fever, enlarged mandibular lymph nodes, depression, ocular and nasal discharge, diarrhea, vomiting, dyspnea, coughing, pneumonia, and neurological signs such as tremors, circling, head tilt and convulsions [2,13,14]. CDV should be considered in any young dog with multisystemic signs and fever. None of the many signs reported in cases are pathognomonic for CDV, rather the clinical picture of CDV varies widely, and depends on many factors such as individual immune status, vaccination history, and virus virulence [15]. Commonly reported signs differ widely and range from respiratory signs such as rhinitis, tracheobronchitis and pneumonia [16], ocular discharge [11] and fever, hemorrhagic diarrhea and seizures [17]. Secondary bacterial infections commonly occur resulting in a host of additional signs such as cough, diarrhea, vomiting and dermal pustules [2].

Vaccination is the principal strategy for protection, and once clinical signs develop, treatment is limited to supportive care. Even in settings where a diagnosis is rapidly reached and a high standard of care is available, CDV has a high mortality rate and is a serious animal welfare issue [2]. 

We report on a CDV outbreak in Chile, and consider the complex dynamics of CDV in FRDs following a natural disaster. This observational study is, to our knowledge, the only documentation of a CDV outbreak in domestic dogs following a natural disaster. The objective was to assess the clinical situation in domestic dogs, to perform screening diagnostics to determine the cause of morbidity and mortality, and to vaccinate against common canine diseases to augment immunity in the population. 

## 2. Experimental Section

### 2.1. Background and Study Site

On February 27, 2010, at 3:34 local time (6:34 GMT), south-central Chile was rocked by a major 8.8 magnitude earthquake and subsequent tsunami causing severe destruction in coastal towns. Dichato is a small fishing village (latitude 36°33'02''S, longitude 72°56'47''W) with approximately 3,057 inhabitants located 73 km from the epicenter [18]. Of the 1,817 registered homes in Dichato prior to the tsunami, 958 (52.7%) were reported destroyed in the disaster, and approximately 1,617 (41.8%) of the residents were relocated to temporary camps [18]. There were no containment facilities for dogs, which inevitably led to an increase in the number of FRDs. 

One month following the disaster, accounts of an unknown cause of mortality in domestic dogs began to circulate. Volunteers from a local animal welfare organization reported seeing in a single day, nineteen dogs in camps with multiple clinical signs including severe and debilitating neurological signs. 

### 2.2. First Visit: April 2010

To investigate the cause of this outbreak, on April 22, 2010 a group of 12 veterinarians, students and volunteers from less affected areas of Chile, travelled to Dichato to offer free vaccination clinics for four days. Dog owners were asked to provide a full history including basic owner contact information and current housing status (displaced to camp or in original home) following the earthquake/tsunami, animal signalment and basic health information on the dogs prior to and following the disaster. The majority of dog owners living in temporary camps that were interested in participating in the clinic were able to locate their dogs at some point over the four days. A full physical exam was performed and blood samples were taken from any dog with no history of vaccination for at least one month. A vaccination was administered by licensed veterinarians in appropriate cases. All data were recorded on a patient medical record.

Selecting the most common signs seen in other natural CDV outbreaks [11,12,16,19], we asked owners if their dogs had exhibited: 1-cough, 2-vomiting, 3-anorexia, 4-diarrhea, 5-ocular or nasal discharge, 6-respiratory signs (*i.e.*, dyspnea, cough), 7-rapid weight loss, 8-lethargy or depression, and 9-tremors or seizures since the earthquake. 

### 2.3. Second Visit: May 2010

Dichato was visited for a second time on May 21, 22 and 23. The objective on this second visit was to document changes since April, to re-vaccinate dogs that had received their first vaccination on the first visit, and to expand the vaccine coverage. The same protocol was followed for animal and owner data collection, physical exams and vaccination as in April. Blood was not collected on the second trip because results from screening diagnostics in April had confirmed the cause of the outbreak.

### 2.4. Blood Collection and Diagnostics

Using standard aseptic techniques and manual restraint, 5 mL of whole blood was collected from the cephalic vein into serum blood collection tubes and maintained upright in coolers for a maximum of eight hours. All samples were centrifuged within 6–8 hours of collection, at 2,000 rpm for 5 minutes for serum harvesting. Based on the suspicion that CDV was the causative agent in clinical cases described in Dichato, we used diagnostic tests for detection of IgM antibodies against CDV. Tests were performed on-site to confirm CDV infection on three animals presenting with life-threatening signs. Remaining serum from these three dogs along with all other serum samples were frozen at −20 °C for later testing in a controlled environment. 

Commercially available in-clinic dot-ELISA kits (Biogal’s ImmunoComb^®^ Antibody Test Kit) with a specificity of 95.5% and sensitivity of 93.1% were used as previously described [20] to determine the concentration of IgM antibody against CDV. Briefly, all assays were performed at temperatures between 20 °C and 25 °C. Using a 12-toothed plastic card, with each tooth corresponding to an individual sample, IgM antibodies from the specimen, if present, will bind to the CDV antigens on the test spots yielding a colour result with the intensity corresponding with the antibody level in the test specimen. Results are expressed as S units on a scale of 0 to 6 corresponding to titres of 0, 1:10, 1:50, 1:250, 1:1250, 1:6250 and 1:12,500 respectively on indirect fluorescence immunoassay antibody test. Any result S≥2 (or titre ≥1:50) was considered a strong positive according to manufacturer recommendations, although results of S1 (or titres ≥1:10) indicate recent exposure to the virus [20]. 

### 2.5. Vaccination

To augment immunity against CDV as an aid in outbreak containment, modified live virus vaccines (Duramune Max 5-4L Dog Vaccine, Fort Dodge Animal Health) protecting against CDV, canine parvovirus, Adenovirus Type 2, parainfluenza, and leptospirosis (serovars Canicola, Grippotyphosa, Icterohaemorrhagiae and Pomona) were administered subcutaneously by veterinarians to any dog greater than 8 weeks of age, with no current vaccination coverage and no obvious signs of fever, illness or disease noted on the physical examination. 

### 2.6. Descriptive Analysis

Information from owners and veterinary examinations were recorded on medical records. These data and CDV results were exported into Microsoft Excel^®^ (Version 2007), and statistical analyses performed with Statistix 8 Analytical Software^®^ and SPSS^®^ (Version 15.0). Descriptive analyses are reported with 95% confidence intervals throughout. 

### 2.7. Risk Factors

Owner variables (relocation following the disaster, and observation of signs of illness in their dogs since the disaster) and dog variables (age, sex, signs of CDV on physical exam, body condition score ≤2.5, rectal temperature >39.5 °C and having no previous vaccination) were analyzed as possible risk factors for having a positive result for CDV. Each variable was individually examined for significance using χ^2^ or Fishers Exact tests for small sample sizes, and all results with *P* < 0.05 were considered statistically significant.

## 3. Results and Discussion

### 3.1. Owner Information and Clinical Observations: April and May Visits

Based on national statistics [18] there were 1,817 houses in Dichato prior to the disaster, and 958 were destroyed in the earthquake and tsunami. Using an average of published estimates for other urban areas of Chile (Viña del Mar: 0.95 [21]; Santiago: 0.76 [22], and Guanaquero: 0.8, Tongoy: 0.9 and La Torre: 1.4 in the Coquimbo region of north-central Chile [23]) of 0.962 dogs per household, we estimate that as many as 921 owned dogs were immediately homeless after the disaster. Of 164 owners, 125 (76.2%) had been relocated to temporary camps.

We examined 142 dogs in April and 66 dogs in May for a total of 208 physical examinations performed. Twenty-four of these dogs were examined on both occasions, giving a total of 184 dogs brought to the clinics by 164 people. At this ratio of dogs to owners, we calculated 1.12 dogs per dog owning household which is consistent with findings from other similar locations in Chile [23]. Of all dogs whose sex was recorded (n = 179), 111 (62.0 ± 7.4%) were males and the sex ratios did not differ significantly over the two visits (χ^2^* = 2.05*, *P* = 0.15). Of these, 24 (35.3%) females and 1 (0.9%) male had been sterilized. Owners of 155 dogs were able to recall the previous vaccination history and only 52 (33.5%) had received at least one CDV immunization.

Of all physical examinations performed in April and May (n = 208), information regarding clinical signs observed by owners in their dog(s) since the disaster, was obtained from 199 medical records (95.6%). Eighty-four owners (42.2%) described between one and eight of the nine CDV signs simultaneously, that we inquired about in their dogs. Those reported from most to least frequent were cough (53.6%), vomiting (34.5%), anorexia (29.8%), diarrhea (26.1%), ocular or nasal discharge (21.4%), lethargy or depression (15.5%), respiratory signs (10.7%), weight loss (9.5%), and tremors or seizures (1.2%). Information from physical exams showed similar findings. Of the 208 physical exams in April and May, 45 dogs had at least one of the nine CDV signs, and veterinarians noted dogs with cough as the most frequent signs.

We examined three dogs in April with severe, late stage neurological signs of CDV including myoclonus and convulsions and were found positive for IgM antibodies to CDV. Although not statistically significant, it was observed that there were more dogs in April with acute respiratory signs, such as severe cough and dyspnea (7.7% *versus* 3% in May) and neurological signs such as seizures, ataxia and myoclonus (5 dogs *versus* no dogs in May). Conversely, in May we observed more chronic illnesses such as bacterial and parasitic dermatitis (37.9% *versus* 9.2% in April), ocular or otic infections (18.2% *versus* 3.5% in April), and traumatic lesions and injuries such as lameness, unrepaired fractures or superficial injuries (4.5% *versus* 2.8% in April). Of all physical exams performed in both April and May, 50.7% of the dogs had at least one clinical abnormality noted by the attending veterinarian. 

### 3.2. CDV Diagnostic Results

Blood was collected for screening diagnostics in April from 106 dogs greater than four months of age with no history of vaccination against CDV within the previous month. Results for two samples were inconclusive, but of the remaining 104 dogs, 54 (51.9 ± 9.6%) were positive for IgM antibodies to CDV at the cut-off value of ≥1:50. Although we refer to positive dogs throughout as those with a titre of ≥1:50, it is noteworthy that we found an additional 41 dogs with S1 values corresponding to a titre of ≥1:10, bringing the total number of dogs with detectable levels of IgM antibodies up to 95 of the 104 (91.4%). Of all dogs with detectable titres (n = 95), 41 dogs (43.2%) had a dilution of 1:10, 33 dogs (34.7%) had 1:50, 17 dogs (17.9%) had 1:250 and 4 dogs (4.2%) had a titre of 1:1250. 

### 3.3. Vaccination in April and May

We vaccinated 128 dogs in April and 49 in May for a total of 177 dogs vaccinated over the two visits. Twenty-one of these dogs were re-immunized (boosters) on the second visit, giving a total of 156 newly vaccinated dogs in the population.

### 3.4. Risk Factors for CDV in Dogs

There was no difference in the positive CDV results between dogs whose owners had been relocated to a temporary camp after the disaster (n = 22, 53.3%) and dogs whose owners stayed in their original homes before and after the disaster (n = 35, 56.5%) (χ^2^ = 0.08, *P* = 0.78). 

Owners reporting signs or generalized illness in their dogs following the disaster appeared to correlate well with the CDV test results: out of the 75 owners that observed one or more of the set of CDV signs in their dogs, 57 of those had a positive result, while all 28 owners reporting no signs of illness in their dogs were negative for CDV antibodies (χ^2^ = 47.65, *P* = 0.001). 

The year of birth was only known for 95 of the dogs that had a CDV result and the highest number of positives (n = 26, 50.0%) was in young dogs and puppies born between 2008 and 2010 (Fishers Exact = 19.4, *P* = 0.02).

The sex of the dog, having a body condition score of less than 2.5, and having clinical signs present or a body temperature higher than 39.5 °C at the time of the veterinary exam were considered as additional risk factors for CDV, but none proved to be significant indicators of dogs having a positive CDV result (χ^2^ = 0.86, *P* = 0.36, χ^2^ = 2.65, *P* = 1.03, χ^2^ = 0.05, *P* = 0.83, and χ^2^ = 2.51, *P* = 0.11 respectively).

Seventy-eight dogs with positive CDV test results had a known vaccine history (78/155; 50.3%). Twenty-seven had been vaccinated at least once previously in their lifetime, and 13 of those were positive (48.1%). Of the 51dogs that had never been vaccinated, 29 were positive (56.9%). There was no difference in CDV seroprevalence among vaccinated and unvaccinated dogs (χ^2^ = 0.74, *P* = 0.39). 

### 3.5. CDV Outbreak in Dichato

Following the February disaster, the majority of the dogs that we sampled in Dichato (51.9%) had titres corresponding with a confirmed exposure to CDV or recent vaccination. Since none of the sampled dogs had been vaccinated at least since the earthquake, we can assume that the source of IgM antibodies was a recent natural exposure [20]. Additional dogs with low titres brought the total dogs having any detectable IgM antibody titres to CDV to 91.4%. Although these results could potentially include dogs that were vaccinated just prior to the earthquake, no owners recalled having their dogs vaccinated within the three months prior. Interestingly, the majority of seropositive dogs were not presenting with major clinical signs of CDV at the time of examination despite the high percentage of dogs with IgM titres. This suggests one of a number of possibilities. Some of the dogs may have been previously ill and had recovered, they could have been incubating the disease at the time of examination, they could have been previously exposed and had mounted an adequate immune response resulting in subclinical infection, or the virus could have been of low virulence. In the absence of pre-disaster baseline data on the prevalence and incidence of CDV or other diseases in this population, it is impossible to make any epidemiological inferences. However we postulate that based on the high proportion of dogs with recently reported illness and clinical signs observed at the time of exam, together with measurable IgM titres in the majority of this population, it is plausible that the onset of this CDV outbreak occurred after the disaster. 

Although our findings support the occurrence of a CDV outbreak in Dichato, we were unable to conduct a full epidemiological investigation enabling us to describe major components of the outbreak such as morbidity and mortality rates. In general, there is poor surveillance and reporting at the global level for CDV. For this reason, veterinary clinics have served as an important resource in other outbreaks to confirm and describe epidemiological findings in pet dogs [16,19]. In Dichato, there were no veterinary clinics or services, no diagnostic or treatment capability, and the loss of inherent infrastructure severely hindered attempts to monitor or control the outbreak in domestic dogs. 

### 3.6. Detecting IgM Antibodies to CDV

IgM antibody tests are reliable in identifying clinical and subclinical infections in recently exposed, vaccinated and unvaccinated dogs with a positive result indicating recent or ongoing viral multiplication [2,12,20,24]. IgM antibodies should be detectable approximately 7–9 days post-infection and maximum values approximately 7–14 days later, with a duration of up to 90 days in total [25], and similar to human measles, any measurable titre of IgM can be considered a recent exposure to the virus [20,26]. 

The dot ELISA method for detecting IgM antibodies to CDV proved useful for screening under disaster conditions. Rapidly available results enable on-site decision making when necessary, and unlike IgG tests in which paired samples are recommended, IgM results for CDV are based on a single test [20]. Tests were cost effective, easy to perform and read, and did not require any sophisticated equipment. The only complication that arose in the field was the requirement for a stable temperature of 20–25 °C while conducting the test. According to historical temperatures for the area, recorded lows during our stay in Dichato ranged between 0 and 5 °C [27] and access to heated areas can be difficult and sporadic in disaster stricken areas. 

### 3.7. Vaccination Status and CDV Outbreaks

Owners of vaccinated dogs with a seropositive result were unable to produce vaccine dates, frequency, or manufacturer information as many had lost all their belongings in the tsunami. However, no dogs had been vaccinated since the earthquake (>54 days) and no owners of vaccinated dogs could recall having their dogs vaccinated in the three months prior to the earthquake. There were no veterinary services offered in Dichato so owners wishing to vaccinate their dogs were required to drive to seek veterinary attention in other towns. Any owners giving highly ambiguous information about vaccination dates were not included in the study. In spite of the lack of information on these dogs, vaccinated dogs yielding positive results for CDV are not entirely rare events during an epidemic. Explanations can vary from being a normal immune response to a natural exposure, waned immunity resulting from inadequate re-vaccination, vaccine failure, or an individual’s inability to mount an appropriate immunological response, but the actual reasons for this phenomenon during outbreaks are rarely confirmed [2,9,16,24]. In the absence of more detailed information on vaccine type and dates and without having knowledge of the IgG serum titres for these animals, it is impossible to determine if any or all of these factors could have played a role in this community of dogs. 

Although vaccination rates are used as convenient indicators of herd immunity (HI), the reproductive rate (R_o_) for CDV has not been measured in field studies, but certainly as with immunity, it will vary according to the spatial structure, age and contact rates of the population and management and behavioral factors [9]—all unknowns for FRD populations. According to owners, only 34% of all the dogs we saw had been previously vaccinated against CDV at least once in their life. This is a similar finding to that of another study in Chile where the vaccination rate was 29% [23]. During an outbreak in India, it was found that all dogs vaccinated more than a year prior to the onset of the outbreak were at equal risk of contracting CDV to unvaccinated dogs [11]. This highlights the importance of maintaining appropriate levels of vaccination in canine populations in promoting herd immunity, and justifies the implementation of vaccination campaigns following disasters to prevent outbreaks of canine diseases in susceptible populations. 

It is surmised that, similar to human measles, protective vaccine coverage against CDV as high as 90–95% may be required [9,12,26]. We introduced 156 newly vaccinated dogs (177 total and 21 booster vaccinations) into the population, which would not be sufficient to raise the herd immunity to a protective level in this population. Although we were equipped with adequate vaccines to accomplish a much higher coverage level, our effort was limited by the number of people interested in the service and during these two visits, there were simply no more owners willing to bring their dogs forward for a vaccine. 

### 3.8. Risk Factors for CDV

We found no relationship between CDV prevalence and the location in which the dog owner was living, suggesting that the dogs in this community could be acting as one large community, potentially connected by the movements of FRDs. The only risk factor for having CDV that we identified in this outbreak was age. The majority of seropositive cases were detected in dogs between 4 and 24 months and this was consistent with findings in other CDV outbreaks and the reported epidemiology of CDV [1,2,8,14,16]. Age-related susceptibility is presumed to decline after 6 months with vaccine-acquired immunity [2], however in communities such as Dichato where vaccination rates are very low, age-related immunity is more likely derived from low-grade non-fatal exposures to endemic diseases within the dog community and may therefore undulate over time depending on the population-wide susceptibility and dynamics.

### 3.9. Impact on FRDs

In the absence of information on how many dogs were previously free-roaming, there is no way to determine the actual impact of FRDs following a disaster. However it is certain that there would have been more FRDs following the disaster due to destruction of homes. We did not do a FRD population estimate in Dichato, however the majority of the owners (n = 125, 76.2%) whose dogs we examined had been relocated to temporary camps. In these camps, there were no means for disposal of animal excrement, no holding facilities for dogs such as kennels or yards and few dogs were seen tied up. Low numbers (36.2%) of the dogs we examined had been sterilized and coupled with the absence of management strategies for FRDs in the aftermath of disasters, uncontrolled reproduction likely increased.

CDV rapidly replicates and disseminates when the immune system is weakened [11,12] and it is known that FRDs suffer from high mortality, malnutrition, starvation, diseases and abuse [6]. Although there is no baseline knowledge of expected IgM titres in this population of free-roaming dogs prior to the earthquake, we assume that the welfare and public health and sanitation effects experienced by the community as a result of large populations of FRDs [6,23,28,29,30,31,32,33,34,35,36] may all be exacerbated following a disaster. Indeed, dogs examined in May had more signs of ectoparasitic infestations, chronic infections and injuries than those examined in April. Admittedly, the numbers of dogs seen in May were lower than those seen in April. However, this finding raised the concern that a prolonged duration of inadequate health care, shelter and nutrition was taking its toll on the quality of life of these dogs.

Future longitudinal studies would shed light on the extent to which disasters play a role in disease outbreaks particularly in FRDs. However, we surmise that this canine population experienced a considerable number of physical and environmental stressors, thereby increasing their vulnerabilities by disrupting the pathogen-host relationship [37], and facilitating the emergence of an endemic disease. Infected dogs permitted to roam freely in urban or rural areas pose a risk to other susceptible individuals by thwarting any attempts of disease containment. 

## 4. Conclusions

CDV is an important disease with serious animal welfare implications for FRDs in the post-disaster setting. The loss of basic infrastructure in the aftermath of disasters affects communities at all levels from national and local governments and health care services [10], down to the animals, their owners and the availability of veterinary services. The possibilities of emergence of other diseases, such as canine zoonoses should be considered. We are hopeful that the information presented here will serve as a starting point for the overdue discussion on risks to the animals themselves following natural disasters. The importance of pre-disaster animal health and preparedness planning, especially where uncontrolled FRD populations exist, continues to be a very real, and unsolved problem [7,38].
